# Timothy syndrome and CACNA1C-Related Disorder: first international language and management guidelines consensus statement

**DOI:** 10.1038/s41431-026-02178-8

**Published:** 2026-07-09

**Authors:** Jack F. G. Underwood, Katherine W. Timothy, Holly Tyroll, Rebecca J. Levy, Ivy E. Dick, Geoffrey S. Pitt, Elizabeth M. Tunbridge, Anwar Baban, Gemma Wilkinson, Nicola A. L. Hall, Georgia Sarquella Brugada, Rosemary Bauer, Dominic Abrams, Jeremy Hall, Timothy Syndrome Alliance (TSA), Sophie Muir, Nick Muir, Meg McLaughlin, Galina Gardiner, Gemma Duncan, Susan Bresnahan, Achsah James, Glenn Miller, Christine Ziegler

**Affiliations:** 1 Neuroscience and Mental Health Innovation Institute, Cardiff University, Cardiff, UK; 2 The Timothy Syndrome Foundation, Charitable Organization, Brigham City, UT USA; 3 Department of Neurology and Neurological Sciences, Stanford University, Stanford, CA USA; 4 Department of Pharmacology and Physiology, University of Maryland, School of Medicine, Baltimore, MD USA; 5 Cardiovascular Research Institute, Weill Cornell Medicine, New York, NY USA; 6 Department of Psychiatry, University of Oxford, Warneford Hospital, Oxford, UK; 7 The European Reference Network for Rare, Low Prevalence and Complex Diseases of the Heart (ERN GUARD-Heart), Amsterdam, The Netherlands; 8 Department of Cardiology, Bambino Gesu Hospital, Rome, Italy; 9 Department Clinical Molecular Genetics and Precision Medicine, Mediclinic City Hospital, Dubai, UAE; 10 Oxford Health NHS Foundation Trust, Warneford Hospital, Oxford, UK; 11 Medical Science Department, School of Medicine, University of Girona, Girona, Spain; 12 Pediatric Arrhythmias, Inherited Cardiac Diseases and Sudden Death Unit, Cardiology Department, Sant Joan de Déu Hospital de Barcelona, Barcelona, Spain; 13 Pediatrics Department, School of Medicine, Universitat de Barcelona, Barcelona, Spain; 14 Department of Cardiology, Boston Children’s Hospital, Harvard Medical School, Boston, MA USA; 15 Hodge Centre for Translational Neuroscience, Cardiff University, Cardiff, UK; 16 Timothy Syndrome Alliance (TSA), Registered Charity, Stroud, UK

**Keywords:** Medical genetics, Neurodevelopmental disorders, Cardiovascular diseases, Neurological disorders, Psychiatric disorders

## Abstract

Timothy syndrome is a multisystemic genetic disorder, classically characterised by prolonged QT interval and subsequent cardiac arrhythmias, neurodevelopmental disorders including developmental delay and autism and syndactyly or hip dysplasia. It is caused by variants in the *CACNA1C* gene, which encodes the widely expressed Ca_v_1.2 voltage-gated calcium channel. Since its characterisation in 2004, the number of variants in *CACNA1C* associated with Timothy syndrome has expanded. With advances in sequencing and the inclusion of *CACNA1C* in genomic screening, further variants have been identified presenting with incomplete features of Timothy syndrome or further aligned phenotypes, which are inconsistent with the original description. In the absence of a formal nomenclature, these presentations have been reported in a proliferation of ill-defined terms, e.g. Atypical Timothy Syndrome. At the same time, advances in knowledge and therapeutics have improved morbidity and life expectancy for these individuals when appropriately identified and managed. Here, we present guidelines for the diagnosis of individuals presenting with variants in *CACNA1C*, developed by an international panel of experts through Delphi consensus with the involvement of the *CACNA1C* community. We formalise the language around syndromic presentations linked to *CACNA1C* variants, reassert and demarcate the classical Timothy syndrome phenotype and provide a structured co-produced definition for CACNA1C-Related Disorder. Finally, we present minimum expected standards of clinical care for individuals with CACNA1C-Related Disorder or Timothy syndrome, with implications for long-term management and improved outcomes for affected individuals.

## Introduction

Timothy syndrome (TS) (Online Mendelian Inheritance in Man (OMIM) entry #601005) is a multisystemic disorder incorporating physical, neurodevelopmental and psychiatric features. It was first described in 1992, through cases of a novel prolonged QT arrhythmia syndrome associated with syndactyly (finger and toe webbing) [1, 2]. It was formally characterised in 2004 through the work of the Keating and Splawski labs [3] and named after Katherine Wilson Timothy (KWT), Clinical Coordinator and key force behind the search and triangulation of the genetic variant underlying TS.

The causative variant for TS was isolated to a missense p.G406R change in the *CACNA1C* gene at 12p13.33, encoding exon 8A of the Ca_V_1.2 L-type voltage-gated calcium channel [3]. Its ultra-rare presentation was coupled with significant early-life mortality, which has been reduced with the uptake of screening for prolonged QT in infants born with syndactyly. Population prevalence remains unknown, with current estimates of <1/1,000,000, classifying presentations as ultra-rare [4]. KWT’s 2024 Natural History Study of TS included 87 cases [5], whilst the Timothy Syndrome Alliance’s (TSA’s) global *CACNA1C* Community Registry includes 104 individuals as of October 2025. Efforts by KWT, the Timothy Syndrome Foundation (TSF) and the Timothy Syndrome Alliance (TSA) to raise awareness continue, with the inclusion of *CACNA1C* in a range of gene-screening panels and clinical implementation of comprehensive non-targeted next-generation (exome or genome) sequencing leading to a rapid increase in individuals joining the patient community.

Since the first description of Timothy syndrome, the spectrum of phenotypes has expanded, as further variants and affected organ systems have been identified. An early advance was the recognition that the thirteen children in whom TS was first identified all had an identical p.G406R variant in exon 8A [3]. Exons 8 and 8A can be alternatively spliced in a mutually exclusive manner and in 2005, a further individual with TS features, most notably an arrhythmia syndrome associated with marked QT prolongation but no syndactyly, was identified [5, 6]. This individual, with a p.G406R variant present in exon 8 and congenital hip dysplasia, was the first of a small number that led to the definition of TS Type II (TS2), differentiated from TS Type I (TS1) in exon 8A. An additional case in the same study, of an individual with a p.G402S variant in exon 8 and with a similar phenotype, was also included in TS2, extending the molecular diagnostic criteria to other variants within *CACNA1C* for the first time [6]. It should be noted that the exon 8/8A nomenclature is inconsistent in the literature, with many early papers referring imprecisely to Timothy syndrome across exons 8 and 8A. Here, we utilise the nomenclature originally described in Splawski *et al*, with TS1 comprising a phenotype including syndactyly and TS2 featuring hip dysplasia [6].

The initial descriptions of TS invariably included prolongation of the QT interval, a feature which was soon classified as Long QT syndrome type 8 (LQT8). Subsequent work focused on individuals with isolated prolonged QT intervals, without any extracardiac TS features [7] such as cognitive impairment or facial dysmorphology [7, 8]. Some reported cases of novel *CACNA1C* rare variants have included symptoms that mirror or physiologically oppose TS or LQT8, such as shortened QT duration or Brugada ECG patterns. These studies, however, are predominantly single cases, lack familial genetic testing pedigrees and often the variants presented occur at relatively common population frequencies. For example, in 2018, the Clinical Genome Resource (ClinGen) re-examined the gene-disease relationship for *CACNA1C* and Brugada Syndrome and classified the evidence supporting a causal role for *CACNA1C* in Brugada Syndrome as limited [9].

**Fig. 1:**
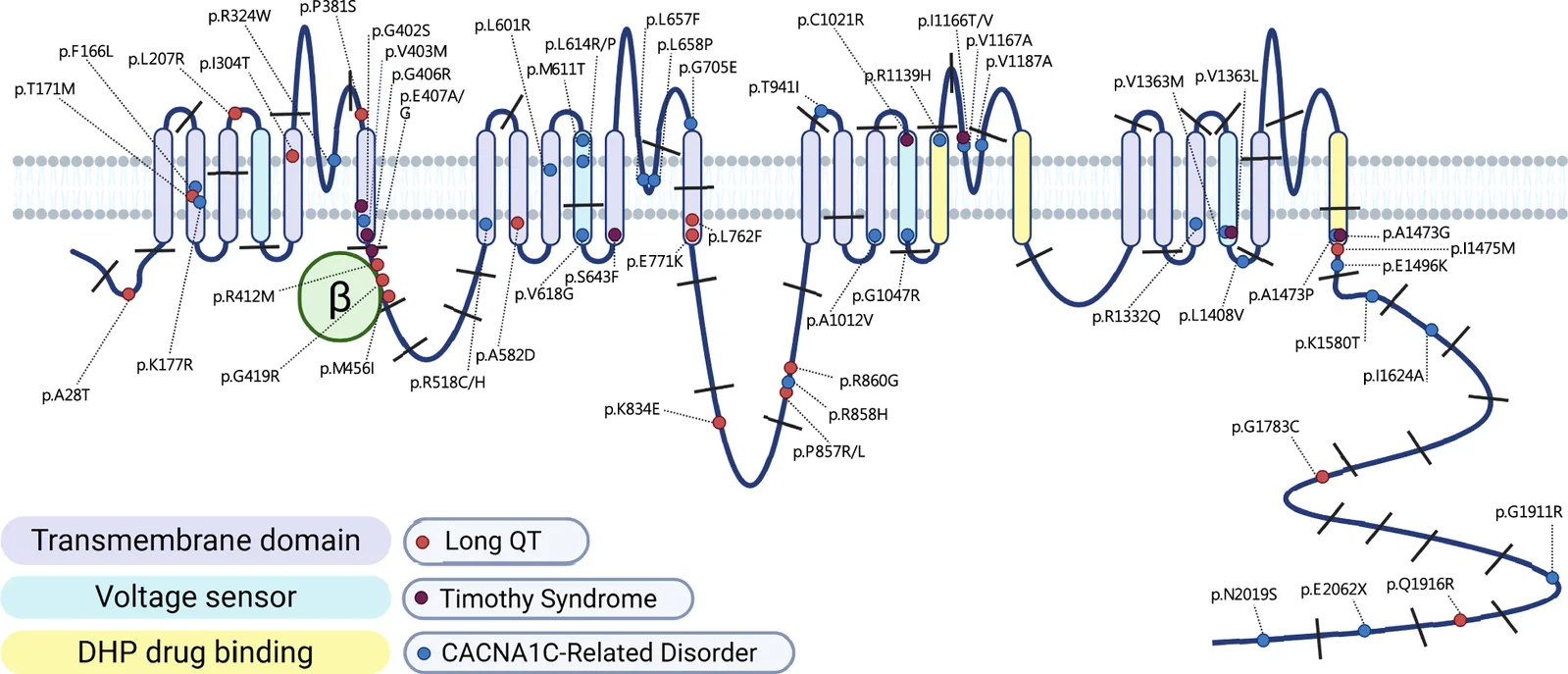
Published variants in CACNA1C mapped to approximate locations within the Cav1.2 protein. Based upon review of published variants undertaken to September 2025. See Supplementary Material for list of variants and publications. Created in BioRender. Underwood, JFG & Tunbridge, E. (2026) https://BioRender.com/kti25xm.

Beyond cardiac presentations, rare variants in the *CACNA1C* gene have also been associated with syndromic presentations in sporadic case series and reports (Fig. 1) [10–14]. These symptoms overlap with the original phenotype ascribed to TS, including some, but not all, features. Neurodevelopmental disorders are frequently observed: developmental delay, intellectual disability, epilepsy, autism, attention-deficit hyperactivity disorder (ADHD) and hypotonia [10, 11, 13, 14]. Efforts establishing this phenotype over the past five years have led it to be added to multiple reference sources, including OMIM (#620029), Orphanet, Wikipedia and Gene Reviews [15]. The largest and most rigorous case series and literature reviews now strongly suggest that rare variants in the *CACNA1C* gene cause a severe multisystemic disorder, but no single review examines all potentially implicated phenotypic features (Table 1).

**Table 1:** Overview of case series and literature reviews examining Timothy syndrome, CACNA1C-Related Disorder and long QT syndrome 8.

	Timothy *et al*, A Natural History of Timothy Syndrome, 2024 [5]	Walsh *et al*, A multicentre study of patients with Timothy syndrome, 2017 [18]	Rodan *et al*, Phenotypic expansion of *CACNA1C*-associated disorders to include isolated neurological manifestations, 2021 [10]	Borbás *et al*, Geno- and phenotypic characteristics and clinical outcomes of *CACNA1C* gene mutation associated Timothy syndrome, ‘cardiac only’ Timothy syndrome and isolated long QT syndrome 8: A systematic review, 2022 [41]	Levy *et al*, A cross-sectional study of the neuropsychiatric phenotype of *CACNA1C*-related disorder, 2022 [14]	Matthews *et al*, International Cohort of Neonatal Timothy Syndrome, 2024 [63]	Cipriano *et al*, Expanding the Phenotype of the *CACNA1C*-Associated Neurological Disorders in Children: Systematic Literature Review and Description of a Novel Mutation, 2024 [13]
Sample size	87	6	25	59	24	44	35
**Prolonged QT interval/ arrhythmia**	Identified	Identified	Identified	Identified	Identified	Identified	Excluded ^b^
**Shortened QT interval**	Not discussed	Not discussed	Not observed	Excluded ^a^	Not discussed	Not discussed	Excluded ^b^
**Intellectual disability**	Not discussed	Not discussed	Identified	Not discussed	Identified	Not discussed	Identified
**Developmental delay**	Identified	Identified	Identified	Identified	Identified	Not discussed	Identified
**Syndactyly**	Identified	Identified	Identified	Identified	Not discussed	Identified	Not discussed
**Hip dysplasia**	Identified	Identified	Identified	Not discussed	Not discussed	Not discussed	Not discussed
**Structural cardiac malformations**	Identified	Identified	Identified	Not discussed	Not discussed	Identified	Identified
**Arrhythmia**	Identified	Identified	Identified	Identified	Not discussed	Identified	Excluded ^b^
**Autism**	Identified	Not discussed	Identified	Identified	Identified	Not discussed	Identified
**ADHD**	Identified	Not discussed	Identified	Not discussed	Identified	Not discussed	Not discussed
**Hypoglycaemia**	Identified	Identified	Not discussed	Identified	Not discussed	Identified	Not discussed
**Hypotonia**	Identified	Not discussed	Identified	Not discussed	Identified	Not discussed	Identified
**Epilepsy/ seizures**	Identified	Identified	Identified	Identified	Identified	Not discussed	Identified
**Immune dysfunction**	Identified	Not discussed	Not discussed	Identified	Not discussed	Not discussed	Not discussed

*Identified* assessed and observed in cases in study, *Not discussed* not assessed in cases in study.

^a^ Borbás et al excluded case reports on short QT syndrome and Brugada syndrome associated with CACNA1C from their review.

^b^ Cipriano et al excluded case reports with ‘documented cardiac conduction defects’.

As the literature on rare variants in *CACNA1C* has expanded, the language and nomenclature used to describe those presentations have become chaotic and confusing. Beyond TS and LQT8, a range of other terms can be found across published literature and public-facing media, including Atypical Timothy Syndrome, Timothy Syndrome Type 3, Timothy Syndrome Variant, Timothy Syndrome-like *CACNA1C* Disorder, Cardiac-only Timothy Syndrome, *CACNA1C*-Related Disorders and *CACNA1C*-Associated Neurological Disorders. These terms have no clear, established or defined clinical meaning. The number of identified individuals with rare *CACNA1C* variants is increasing, support groups and communities are growing and therapeutic treatments are in development [16]. There is therefore a need for clarity to guide families, researchers and clinicians. In this work, we set out to achieve cohesion through convening an international network of experts and engaging with the leading TS and *CACNA1C* charities. This resulting co-produced consensus document outlines a standard language definition for individuals presenting with variants suspected to be pathogenic in *CACNA1C*, along with the guidelines for their diagnosis and management.

## Methods

Development of the *CACNA1C* consensus guideline was modelled on the Delphi Process [17], incorporating multiple rounds of expert discussion, followed by engagement and co-production with the TS and *CACNA1C* community. The *CACNA1C* language consensus group consisted of twelve participants from ten institutions across the USA, UK and Europe, comprising clinicians, clinical scientists and researchers. Discussions occurred via video conference calls, e-mail communications and draft file outcomes exchanges.

**Fig. 2:**
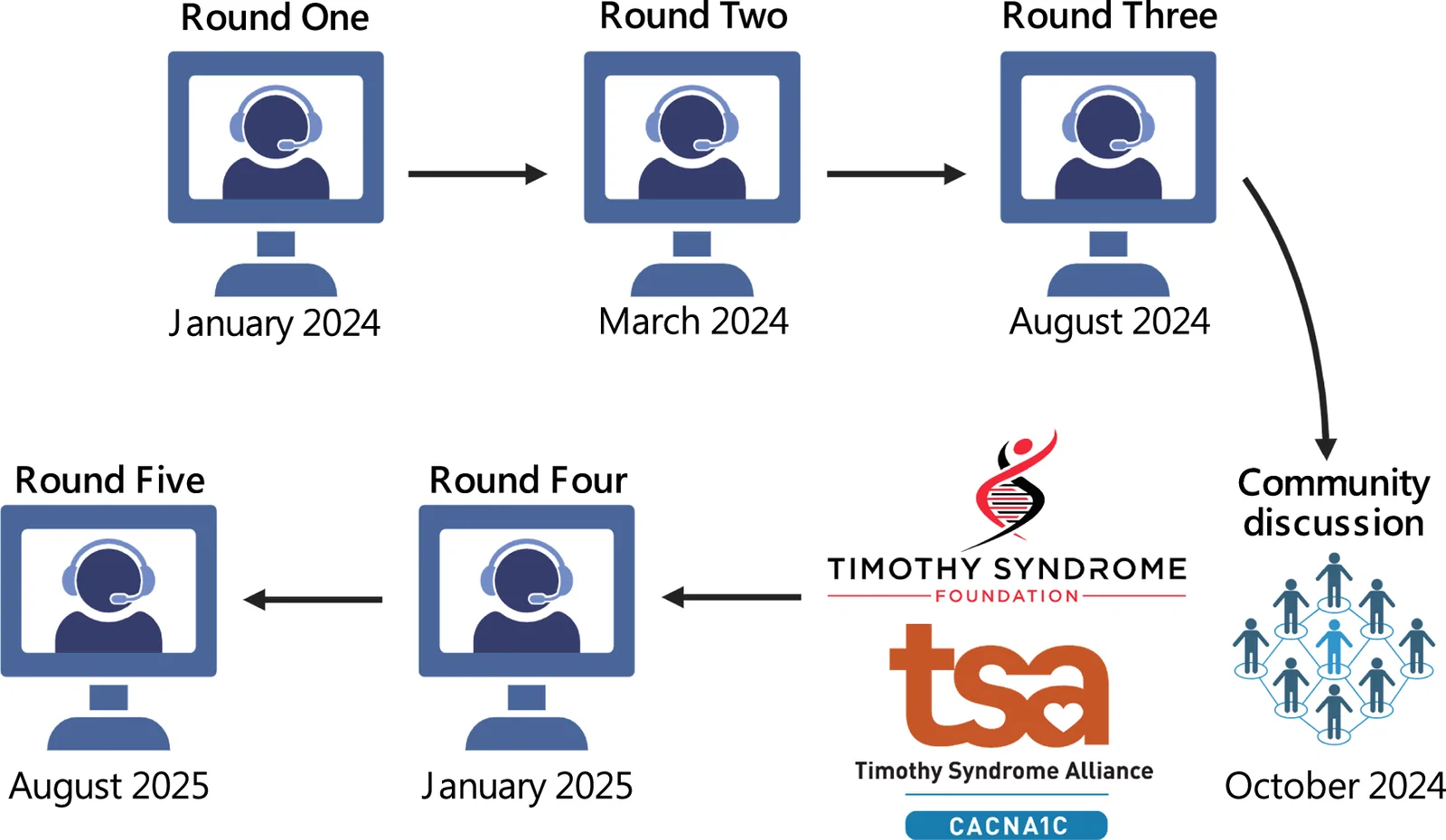
Timeline of meetings and public engagement of the *CACNA1C* language consensus group. This figure was created in BioRender. Underwood, J. (2026) https://BioRender.com/etvxcc3.

Key issues for discussion were initially identified following the *Connect CACNA1C Global Network Conference* held by the Timothy Syndrome Alliance (TSA) in June 2023. The language consensus group was convened and after three rounds of discussions from January to August 2024 (Fig. 2), language options were presented to the TS and *CACNA1C* community for input, along with identification of any further relevant issues. Individuals with *CACNA1C* variants and their caregivers were contacted through social media, support groups and the Timothy Syndrome Alliance (TSA) community mailing lists, reaching >200 families. The community voted on language options, which were then discussed along with further outcomes to generate consensus recommendations at a further two working group meeting rounds.

### Clinical diagnostic criteria

#### Clinical features of Timothy syndrome

As noted, TS Type 1 was defined through cardinal features of prolonged QT interval, cardiac arrhythmia and syndactyly in individuals with a p.G406R gene variant in exon 8A of *CACNA1C* [3]. Further features included autism, developmental delay, seizures, baldness at birth, characteristic facies (flattened nasal bridge, low-set ears, small upper jaw, thin upper lip), small teeth, hypoglycaemia, hypothermia and hypotonia [3, 18]. Inclusion of individuals with p.G406R variants in exon 8 associated with a similar phenotype but featuring hip dysplasia and lacking syndactyly led to the definition of TS Type 2 [6]. These remain the classical signs of TS Type 1 (hereafter TS1) and Type 2 (TS2) and our consensus reaffirmed the importance of these diagnostic entities. The p.G406R gene variant has been recurrently identified among unrelated affected individuals, with the only known unaffected individual ascertained to be mosaic after the p.G406R variant was inherited and identified in their affected child and we therefore expect individuals with p.G406R variants to present with symptoms of TS1 or TS2, dependent on whether the p.G406R variant is present in exon 8 or 8A [12, 15].

**Fig. 3:**
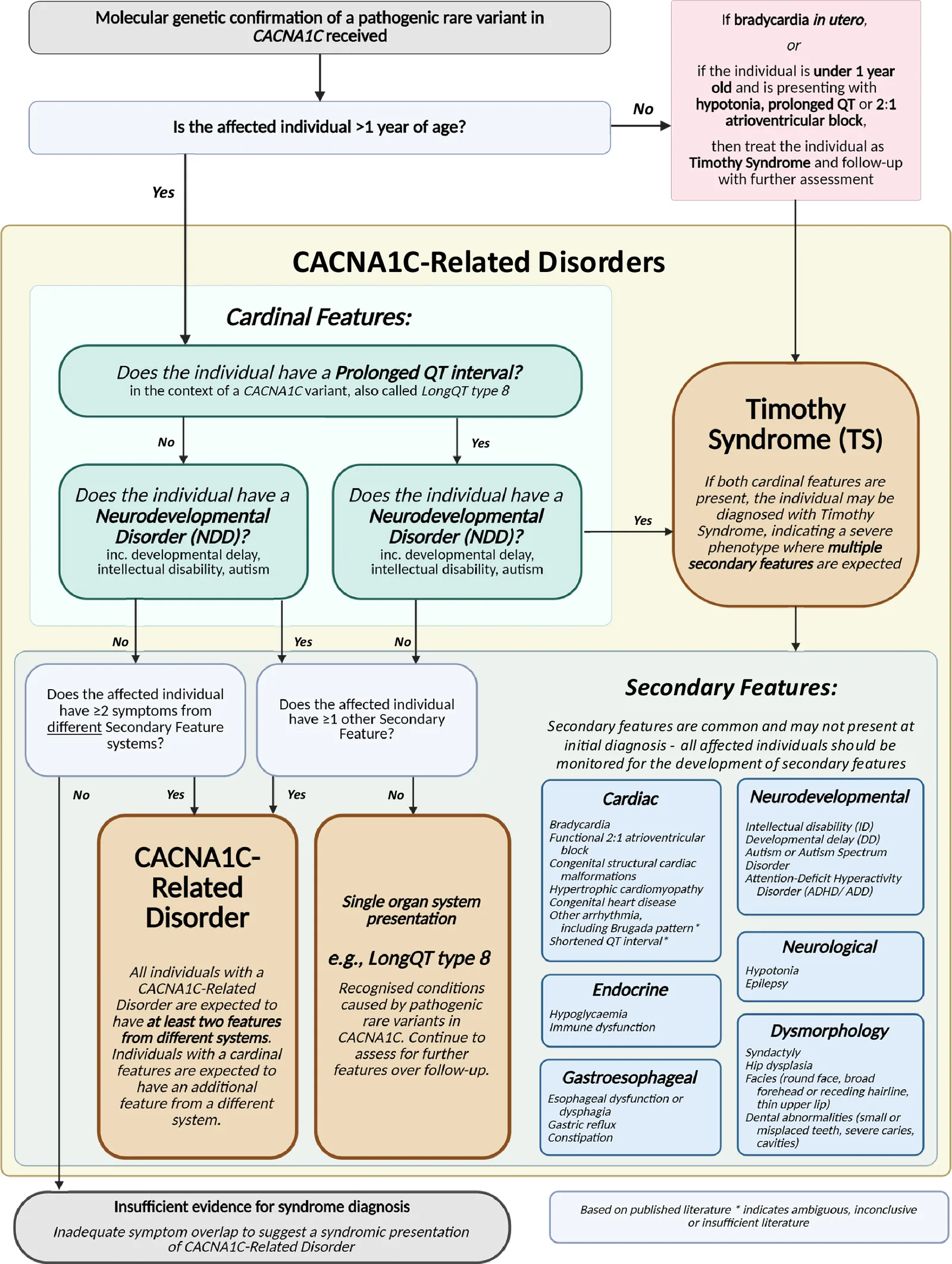
Flow chart for syndromic interpretation and diagnosis in individuals with pathogenic *CACNA1C* rare variants. This figure was created in BioRender. Underwood, J. (2026) https://BioRender.com/va34rza.

In Splawski *et al*’s work defining TS2 they included p.G402S variants [6] and further case reports and series have presented individuals with the classical phenotypic features of TS but novel de novo gene variants, notably p.E407G [19], p.E407A [20], p.C1021R [21, 22], p.I1166T [7, 20, 23] and p.A1473G [24]. On this syndromic basis, TS1 and TS2 diagnoses should be assigned based on phenotypic syndromic features and not be dependent upon the specific p.G406R variant. Any individual with a rare single-nucleotide variant within the *CACNA1C* gene presenting with the classic dyad of prolonged QT interval and a neurodevelopmental disorder (e.g. developmental delay, intellectual disability, autism) should be diagnosed as TS following appropriate clinical genetic medical evaluation. In Fig. 3 below, we outline a flow chart for diagnostic interpretation of *CACNA1C* pathogenic rare variants based upon assessment and examination of all potential syndromic features. In order to aid clarity, we recommend providing the Timothy syndrome diagnosis along with the exact protein and genomic change, for example, Timothy syndrome, p.G406R (HG38: g.2504944 G > A). The rationale for the inclusion of genomic coordinates is discussed as part of the Molecular Diagnostic Criteria below.

These individuals should be further assessed across all potentially affected organ systems, with ongoing follow-up on an annual basis to monitor for further symptoms driven by developmental changes. Of note, individuals with pathogenic variants in *CACNA1C* may present solely with bradycardia whilst *in utero* and in the first year of life with functional 2:1 atrioventricular (AV) block (whereby alternate supraventricular impulses cannot conduct as the ventricle remains refractory secondary to QT prolongation) or hypotonia, prior to the development of other features. Neonates or infants may show prolonged QT intervals on electrocardiography, with or without functional 2:1 atrioventricular block. All individuals identified *in utero* or in the first year of life displaying these features should be treated as suspected TS and followed up closely with proactive intervention and management.

### CACNA1C-Related Disorders

**Fig. 4:**
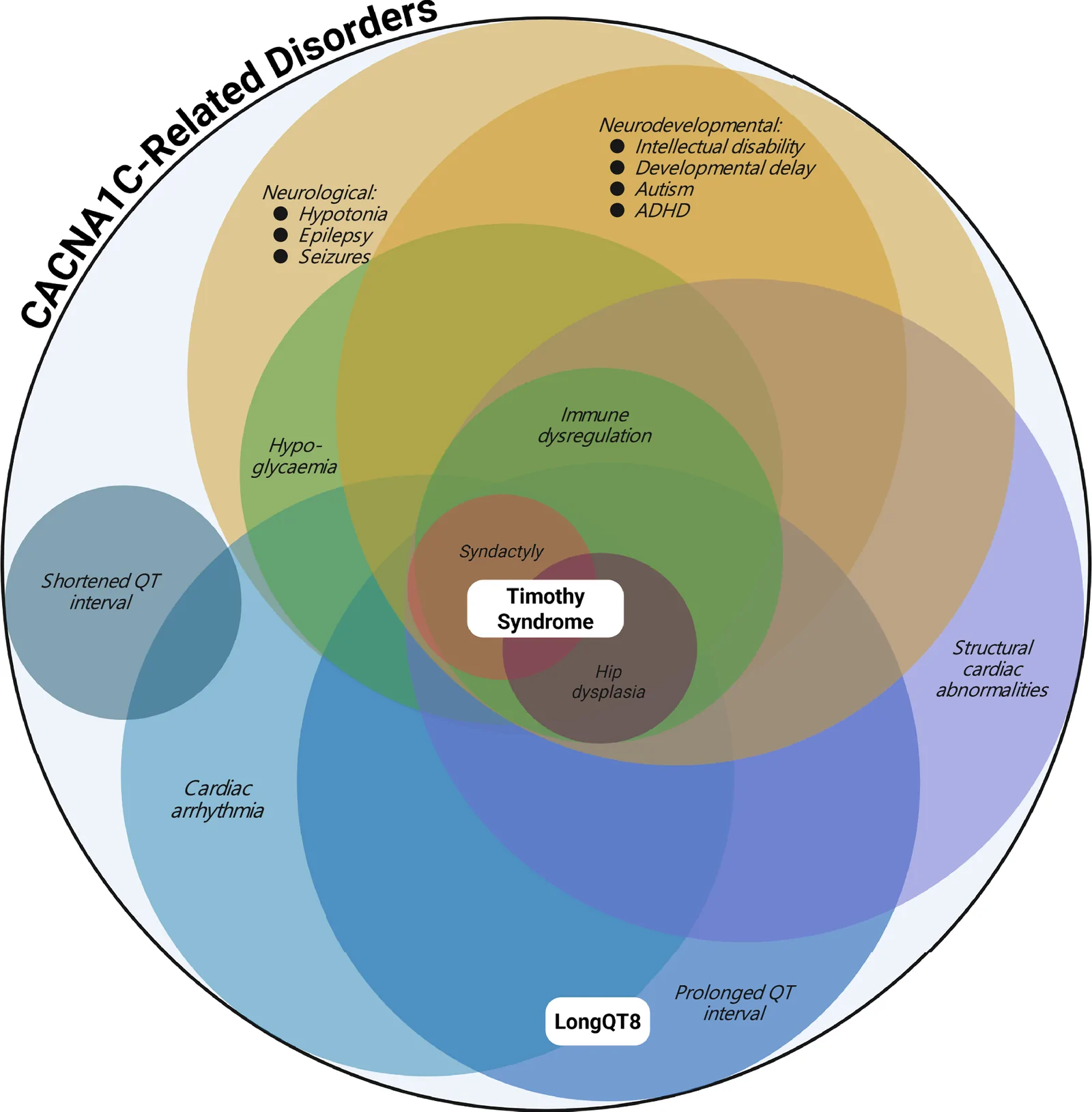
Euler diagram of diagnoses and syndromes featuring *CACNA1C* gene variants. This figure was created in BioRender. Underwood, J. (2026) https://BioRender.com/x2ahbnz.

Individuals with other non-G406R variants in the *CACNA1C* gene may also present with some, but not all, features of TS [10, 11, 13, 14, 25]. Potential diagnostic and nomenclature structures were distilled to two options through the language consensus working group meetings, which were presented to the *CACNA1C* community. 31 individuals with *CACNA1C* variants or their families responded to requests for input, voting by 26 to 3 for the adoption of CACNA1C-Related Disorders terms (with two individuals suggesting further options). This term provides an umbrella grouping for all individuals with pathogenic rare variants in *CACNA1C* beyond those with TS1 or TS2 phenotypes, as laid out in Figs. 3 and 4. This serves to bring those with LQT8 into the wider community and to include those individuals who previously did not have a formal diagnosis, allowing them improved access to medical care and therapies.

Within the CACNA1C-Related Disorders (CRDs) umbrella, syndromic presentations of pathogenic *CACNA1C* rare variants with multiple organ system involvement fitting published literature but inconsistent with TS can be diagnosed with CACNA1C-Related Disorder (CRD) (Fig. 3). CRD has been previously used as a term for individuals with non-TS *CACNA1C* variants across Gene Reviews and previous publications [11, 14, 15], though without agreed descriptors or delineation. Here, through a lived experience co-produced expert consensus method, we provide a formal definition for CACNA1C-Related Disorder. CRD is defined based upon presentations with syndromic features (as identified from established literature) from two different organ systems (Fig. 3), where the individual does not present with the classical dyad of prolonged QT and neurodevelopmental disorder present and consistent in all Timothy syndrome cases. A symptom-based phenotype approach should be taken to assess for diagnosis, given that the underlying pathological mechanism linking *CACNA1C* variants to syndromic outcomes remains unclear.

CRD aligns the *CACNA1C* gene with the movement towards gene-based nomenclature, which has been observed across the field and particularly in channelopathies, e.g. CACNA1A-related disease [26], SCN2A-related disorders [27] and SCN8A-related disorders [28]. In keeping with the suggestions for Timothy syndrome and to provide a clearer disease description for clinicians, we suggest providing diagnoses in a tiered format, whereby the overall CACNA1C-Related Disorder nomenclature is included as a top-level term, followed by a phenotypic description and the exact protein and genomic change, e.g.;

- CACNA1C-Related Disorder
- Developmental delay and hypotonia phenotype
- p.Leu601Arg (HG38 g.2567701 T > G).

#### Cardiac presentations of CACNA1C-Related Disorders

Individuals may initially present with isolated cardiac features, encompassing a range of phenotypes featuring isolated prolonged QT interval, arrhythmias, hypertrophic cardiomyopathy, congenital heart disease, sinus node disease and congenital structural cardiac abnormalities (Fig. 3). The symptomatic presentation of Long QT syndrome type 8 in individuals with rare *CACNA1C* variants has been robustly demonstrated [5, 12, 29–31]. LQT8 has been observed with pathogenic variants distinct from TS, for example, p.P857R in Boczek *et al*’s study of 15 members of a multi-generational family [7] and p.R858H in Gardner *et al*’s study of 26 individuals from a family across five generations [8]. Systematic screening of individuals in long QT databases yielded further case series and novel variants amongst cohorts internationally [23, 32]. The Long QT syndrome type 8 term originates during a period when Long QT syndromes were being sequentially numbered based upon chronological identification. As the understanding of physiology and underlying genomics has improved, other numbered Long QT syndromes are being superseded with gene-based nomenclature. We would suggest that in future consideration be given for the use of Long QT syndrome 8 within the structured format given for CACNA1C-Related Disorders, where diagnosis may be given as CACNA1C-Related Disorder, Long QT Syndrome (LQTS). For consistency, LQT8 will continue to be used through this work.

Many existing case reports on individuals with LQT8 are limited to only cardiovascular symptom profiling and do not include long-term follow-up evaluation, complicating the assessment of a cardiac-specific phenotype. Such studies reporting extra-cardiac features were further complicated by cardiac arrest or hypoxic brain injuries, causing neurological injury secondary to the primary arrhythmias. Concern was raised throughout the consensus group meetings that this may represent under-reporting or under-assessment of non-cardiac phenotypic features. These single organ-system presentations may be due to splice variation, mosaicism or decreased penetrance, but all individuals presenting in such a manner require a full, holistic, multisystemic medical work-up. Individuals presenting with single organ system presentations of a pathogenic *CACNA1C* rare variant (Fig. 3), for example, LQT8, fall within the CACNA1C-Related Disorders umbrella term. Individuals identified via single organ-system presentations early in childhood may present later in development with further symptoms as *CACNA1C* expression regulation changes [5] and therefore ongoing observation and follow-up are advised. In those with *CACNA1C* variants, each individual’s QT intervals can be highly variable with wide ranges and cardiac risk does not clearly correlate with observed QT duration [8, 21]. The emergence of symptoms dependent on developmental stage further supports the umbrella term of CACNA1C-Related Disorders for all patients presenting with a pathogenic *CACNA1C* variant and the need for regular monitoring as standardised baselines of care.

Further cardiac phenotypes have previously been linked with *CACNA1C* rare variants, including Brugada Syndrome (BrS) [33–35] and short QT interval or syndrome (SQT) [33, 36–38]. Shortened QT intervals (SQT) and Brugada Syndrome (BrS) were first reported in individuals with *CACNA1C* p.A39V and p.G490R variants in 2007, not long after the definition of TS. The evidence for such variants as a genetic aetiology of SQT and BrS is disputed [9, 36, 39]. As with LQT8, subsequent case series and reports drawn from clinical cohorts of individuals with established BrS or early repolarisation syndromes have proposed a number of variants across the *CACNA1C* gene linked to a multisystemic phenotype [33, 37, 40]. Reviews of these genes dispute the causal relationship [39], as further evidenced by ClinGen’s re-evaluation of the literature [9]. Subsequent international meetings held on topics of ultra-rare cardiac diseases have supported this opinion, based on insufficient case-level data and lack of functional validation evidencing an association between *CACNA1C* and Brugada Syndrome. We therefore endorse that Brugada Syndrome is not associated with *CACNA1C* variants.

Congenital heart disease and cardiomyopathy presentations are increasingly reported to be associated with *CACNA1C* variants [41–43]. Published cases report cardiomyopathy (hypertrophic or noncompaction) [31, 42–45], congenital heart disease and sinus node disease [7, 42, 46], frequently overlapping with existing documented cardiac features. Where features extend beyond prolonged QT to include other cardiac disease but do not involve other organ systems, the description cardiac-only TS, which was initially an alternative to LQT8, is now also used [29, 30, 43]. We recognise that there is no consensus on this evidence, with a need for further in-depth assessments and functional analyses of posited variants.

### Molecular diagnostic criteria

#### Functional interpretation

The initial novel missense p.G406R variant first identified in exon 8A results is a gain-of-function (increased Ca^2+^ influx during an action potential), manifesting as a lower threshold for channel opening, reduced voltage-dependent inactivation (VDI) and reduced calcium-dependent inactivation (CDI) [3, 6, 47, 48]. Electrophysiological studies have demonstrated that this combination of effects is not isolated to the p.G406R variant [12, 48]. The TS phenotype appears associated pathophysiologically with specific changes in the electrophysiological properties and functions of the Ca_v_1.2 channel, as well as upon how broadly the variant is expressed due to variable mosaicism and/or tissue-specific expression of alternatively spliced exons, as recently reviewed by Bauer *et al*. [12]. Subsequent work has shown that these variants have further effects in neuronal models, including decreased synaptic inhibition, defects in cortical differentiation, altered long-term potentiation and altered cortical interneuron migration [49–51]. Variant function can therefore be understood to be more complex than simple ‘gain- or loss-of-function’ and many variants result in changes of mixed effects at the channel, cellular and system level [7, 12, 23, 25, 48]. Because of this complexity, the current understanding of how variants causative of Timothy syndrome result in clinical pathology is incomplete. Timothy syndrome cannot be adequately explained or defined by known Ca_v_1.2 channel gating changes, as the extant literature covering such an approach does not capture the variability of clinical presentations and therefore Timothy syndrome should be identified through syndromic phenotype.

Further complicating this picture is evidence of both non-linear relationships between *CACNA1C* variants and Ca_V_1.2 expression profiles [47]. Variants may show increased cell surface expression due to decreased degradation [7, 32], while some variants (notably p.G406R) increase basal transcription through excitation-transcription coupling [52] or alter neuronal gene expression [53], with enhanced activation of the Ca_v_1.2 channel driving multiple pathological mechanisms at both the cellular and network level [47, 54, 55]. Expression of Ca_v_1.2 and development of symptoms at a system or organ level are subsequently dependent on transcription of the pathological variant. Ca_v_1.2 is widely expressed in almost all tissues and is critical for development [3, 5, 56]. Evidence from long-read sequencing suggests that *CACNA1C* incorporates at least 47 constituent exons, with >240 novel transcripts and those transcripts demonstrate developmental stage and tissue-specific splicing, which are predicted to alter channel function [57]. These mechanisms, in combination with germline mosaicism, are hypothesised to drive the irregular penetrance and phenotypic variability seen across CRDs [5, 12, 13, 18, 21].

Due to this complexity, clinical pathogenicity should not be extrapolated based upon inferred gain- or loss-of-function. Variants have historically been reported against different *CACNA1C* transcripts, with no single reference transcript existing. This can result in the reporting of alternative coding DNA (c.) and protein (p.) level changes for the same genomic co-ordinates, complicating cross-comparison and interpretation. We therefore suggest that all variants are reported with both the transcript used (for coding DNA and protein changes) and the genomic coordinates. We recommend supporting clinical variant interpretation with functional testing of the variant in established model systems. Tools and literature supporting variant interpretation are predicted to evolve and therefore we advise periodic re-testing and re-evaluation utilising updated evidence. This guidance supports the recommendation that diagnoses should be made on a syndromic basis and pathogenicity cannot yet be inferred from gain- or loss-of-function descriptors alone.

### Guidelines for clinical care

Due to the widespread but variable expression and mixed effects of *CACNA1C* gene variants, presentations are individual-specific but frequently multisystemic. All individuals with a *CACNA1C* pathogenic or likely pathogenic variant require a full multidisciplinary team clinical work-up, to include, at a minimum, cardiac, neurodevelopmental, musculoskeletal and endocrine reviews. We expect individuals to show development of new syndromic features and associated risk over time, secondary to changes in expression of *CACNA1C* and the Ca_v_1.2 channel over the lifespan, necessitating consistent follow-up. For individuals with variants of unknown or uncertain significance (VUS) in the *CACNA1C* gene, it is reasonable to clinically screen for other organ involvement to determine if there is additional clinical evidence of a CRD.

Cardiac features of CRDs, including TS, as the most reported and historically most lethal phenotypic presentation, have received significant therapeutic study [5, 12]. Recent reviews and case series have demonstrated that implantable cardioverter-defibrillators (ICDs) are the most effective current treatments for Timothy syndrome and Long QT presentations [18, 21, 29, 58]. Beta-blocker medications have previously been used for cardiac management, but may not prevent arrhythmias or sudden cardiac death and therefore are not recommended as standalone therapy [8, 18, 21]. Surgical interventions, through left cardiac sympathetic denervation to attenuate heterogeneous sympathetic myocardial innervation, have been trialed with some mixed evidence in those with symptoms refractory to other treatments [5, 21, 59]. In individuals whose age- and sex-adjusted QT interval borders on prolonged, some evidence suggests exercise stress testing may precipitate the functional 2:1 AV block and support diagnosis [32, 60]. Calcium-channel blockers, perhaps counterintuitively, are not effective in the management of TS due to the complex gating changes in Ca_v_1.2 [5, 48, 61]. Implantation of an ICD early in life in TS children decreases mortality risk and this change in practice has led to a dramatic increase in life expectancy of TS children since the syndrome was first described [5, 18, 21, 61].

Very few organ-level reviews have been undertaken in TS or more broadly across CRDs. Timothy *et al*’s 2024 Natural History Study of TS covers a broad range of phenotypic features in depth, including discussion of key areas for clinical concern [5]. As TS and CRD children survive longer, there is evidence of emerging mortality risks from further syndromic features [5, 14, 15]. Epilepsy is prevalent across CRDs and seizures may be refractory to standard treatments [10, 11, 13, 14, 25]. Individuals with CRDs where there is suspicion of seizure activity should have neurologic evaluation and seizure rescue medication available in their home, work or school environments. Hypoglycaemia has been noted in TS and CRDs [3, 5, 6, 21] and recent work has demonstrated that this is through dysregulated glucose homeostasis, absent hyperinsulinism and defects in glucagon secretion [62]. Individuals with *CACNA1C* variants are at risk of hypoglycaemia, particularly when in states of physiological stress, including viral illnesses, which may be compounded by arrhythmias and lower seizure thresholds to increase the risk of sudden death [10, 21]. Those with Timothy syndrome and more severe presentations of CRD should therefore monitor blood glucose levels during periods of physiological stress or illness and have rescue kits (e.g. glucose tablets or gel, fruit juice or sugary drinks/candy) available for use in episodes of low blood sugar.

Further features of CRDs will require long-term therapies and interventions and are treated symptomatically and individually. Responses to anaesthesia are reportedly abnormal and care should be taken with all sedation [5, 21]. Many children with CRDs have frequent infections, likely due to altered immunological function [3, 5]. Abnormal dentition, frequent cavities and small displaced teeth requiring surgical intervention have commonly been reported [3, 5, 13]. Gastroesophageal reflux, frequent vomiting and congenital gastrointestinal defects have been reported across CRDs [5, 22]. Chronic constipation is seen in >80% of individuals with TS and anecdotally stated to be a significant issue amongst CRDs [3, 5]. The majority of children with CRDs will experience some developmental delay, particularly of speech [5, 13, 14]. Autism is a highly penetrant phenotypic feature across CRDs and other neurodevelopmental conditions (e.g. ADHD), neurosensory and neuromuscular features such as hypotonia complicate this presentation [5, 10, 14]. All children with CRDs should be provided with individualised, tailored support in education and work environments.

Long-term evolution of features and outcomes, particularly in those without prolonged QT, is not known, although several individuals are now well into mid-life with a range of functional outcomes [5, 14]. Based on the above guidance, minimum standards of care for individuals with TS and CRDs are laid out in Table 2.

**Table 2:** Guidelines for minimum standards of clinical care for Timothy syndrome and CACNA1C-Related Disorder (including CACNA1C-Related long QT syndrome) and supporting evidence.

*Timothy syndrome*		CACNA1C-Related Disorder and LQT8	
Recommendation	Source of evidence	Recommendation	Source of evidence
Annual cardiac review including electrocardiography (ECG/EKG)	1	Annual cardiac review including electrocardiography (ECG/EKG)	1
Consider early insertion of implantable cardioverter-defibrillators (ICDs)	1	Case-specific use of ICDs and loop recorders in those with borderline or prolonged QT interval (age and sex adjusted)	1
Annual neurodevelopmental reviews whilst <18 years of age	1	Annual neurodevelopmental reviews whilst <18 years of age where evidence of developmental delay, neurodevelopmental or neurological features	1
Periodic home ambulatory EEG to identify potential seizure activity, with review by neurologist	2	Neurological assessment including EEG where evidence of developmental delay, neurodevelopmental or neurological features	2
Home glucose monitoring, hypoglycaemia and seizure rescue kits with appropriate training.	2	Family/ general practitioner annual review to include endocrine, neurological and cardiac health status	3
Annual endocrine and neurology review	3		

Where the source of evidence is categorised as 1—multiple case series, 2—single case series and/or multiple case reports, 3—single case reports and/or expert consensus opinion.

## Conclusions

In the thirty years since Keating *et al* first described a ‘heart-hand’ syndrome and twenty years since this was formalised as Timothy syndrome, the landscape of phenotypic features associated with rare pathogenic variants in the *CACNA1C* gene has changed substantially. TS remains the most severe presentation, but genomic variants linked to this syndromic phenotype have extended beyond the classical p.G406R description. Meanwhile, a range of cases have been reported with overlapping or contrasting phenotypic features and novel rare *CACNA1C* variants. Here, through expert consensus and community engagement, we summarise updated phenotypes and features for TS and its associated presentations. We provide a formal definition for CACNA1C-Related Disorder, a syndromic phenotype capturing non-classical and newly recognised presentations of rare *CACNA1C* variants. Finally, we present the first guidelines on standards of diagnostic and clinical care for individuals presenting with a rare *CACNA1C* gene variant, driven by the evidence that, with appropriate interventions, these individuals can live longer, fulfilling lives.

## Supplementary information

Supplementary Material
